# Engineering glutathione biosynthesis of *Saccharomyces cerevisiae* increases robustness to inhibitors in pretreated lignocellulosic materials

**DOI:** 10.1186/1475-2859-12-87

**Published:** 2013-10-01

**Authors:** Magnus Ask, Valeria Mapelli, Heidi Höck, Lisbeth Olsson, Maurizio Bettiga

**Affiliations:** 1 Department of Chemical and Biological Engineering, Industrial Biotechnology, Chalmers University of Technology, Gothenburg SE-41296, Sweden; 2 Current affiliation: Lehrstuhl für Biotechnologie, RWTH Aachen University, Aachen D-52074, Germany

**Keywords:** Lignocellulose, Bioethanol, Inhibitors, Robustness, Glutathione, Redox metabolism

## Abstract

**Background:**

Production of bioethanol from lignocellulosic biomass requires the development of robust microorganisms that can tolerate the stressful conditions prevailing in lignocellulosic hydrolysates. Several inhibitors are known to affect the redox metabolism of cells. In this study, *Saccharomyces cerevisiae* was engineered for increased robustness by modulating the redox state through overexpression of *GSH1, CYS3* and *GLR1*, three genes involved in glutathione (GSH) metabolism.

**Results:**

Overexpression constructs were stably integrated into the genome of the host strains yielding five strains overexpressing *GSH1, GSH1/CYS3, GLR1, GSH1/GLR1* and *GSH1/CYS3/GLR1*. Overexpression of *GSH1* resulted in a 42% increase in the total intracellular glutathione levels compared to the wild type. Overexpression of *GSH1/CYS3, GSH1/GLR1* and *GSH1/CYS3/GLR1* all resulted in equal or less intracellular glutathione concentrations than overexpression of only *GSH1*, although higher than the wild type. *GLR1* overexpression resulted in similar total glutathione levels as the wild type. Surprisingly, all recombinant strains had a lower [reduced glutathione]:[oxidized glutathione] ratio (ranging from 32–67) than the wild type strain (88), suggesting a more oxidized intracellular environment in the engineered strains. When considering the glutathione half-cell redox potential (E_hc_), the difference between the strains was less pronounced. E_hc_ for the recombinant strains ranged from -225 to -216 mV, whereas for the wild type it was estimated to -225 mV. To test whether the recombinant strains were more robust in industrially relevant conditions, they were evaluated in simultaneous saccharification and fermentation (SSF) of pretreated spruce. All strains carrying the *GSH1* overexpression construct performed better than the wild type in terms of ethanol yield and conversion of furfural and HMF. The strain overexpressing *GSH1*/*GLR1* produced 14.0 g L^-1^ ethanol in 48 hours corresponding to an ethanol yield on hexoses of 0.17 g g^-1^; while the wild type produced 8.2 g L^-1^ ethanol in 48 hours resulting in an ethanol yield on hexoses of 0.10 g g^-1^.

**Conclusions:**

In this study, we showed that engineering of the redox state by modulating the levels of intracellular glutathione results in increased robustness of *S. cerevisiae* in SSF of pretreated spruce.

## Background

In order to cope with the stressful conditions in industrial fermentations, robust microorganisms are needed [1]. This applies especially for the production of bioethanol from lignocellulosic feedstocks such as agricultural and forest residues. The recalcitrant nature of these materials requires harsh pretreatment methods, which aside from imposing structural changes facilitating enzymatic hydrolysis generate compounds acting as inhibitors for cellulolytic enzymes as well as microorganisms [2]. Apart from organic acids and phenolic compounds, the furan aldehydes 5-hydroxymethylfurfural (HMF) and 2-furaldehyde (furfural) stand out as particularly challenging for viable production of lignocellulosic ethanol [3]. These compounds have been shown to inhibit several enzymes in glycolysis, decrease specific ethanol production rate, affect cell growth and survival and to induce the formation of reactive oxygen species (ROS) in *Saccharomyces cerevisiae*, with DNA and membrane damage as a consequence [4-7]. Under anaerobic conditions, HMF and furfural are converted *in situ* to less toxic alcohols by NAD(P)H-dependent oxidoreductases [8,9]. Both the detoxification of ROS and the conversion of HMF and furfural *per se*, create a more oxidized intracellular environment by draining the cells of reducing power in terms of NAD(P)H, and potentially obliterate the antioxidant defense system of the cell [10].

Based on these evidences, we hypothesized the intracellular redox system as a potential metabolic engineering target for increasing robustness of *S. cerevisiae* to lignocellulosic inhibitors.

Glutathione (GSH) is the main antioxidant system in living cells and has been shown to be indispensable for, but not limited to, oxidative stress responses [5,11,12]. By virtue of its high intracellular concentration and low redox potential (-240 mV), GSH has been regarded as a biological redox buffer maintaining redox homeostasis in spite of insults caused by oxidizing agents [13]. Its many functions include: scavenging of ROS, protection against endogenous toxic metabolites, detoxification of xenobiotics and involvement in sulfur and nitrogen metabolism (reviewed in [14]).

GSH is a tripeptide composed of cysteine, glutamate and glycine, in which the thiol (-SH) group of the cysteine residue confers the activity to the molecule [14]. GSH occurs intracellularly in either its reduced form (GSH) or in its oxidized form (GSSG) where two GSH molecules are interlinked with a disulfide bond. Utilization of GSH results in the oxidation to its disulfide form, GSSG, from which GSH can be regenerated by the action of glutathione reductase, encoded by *GLR1*[12]. Glr1p uses NADPH as reducing equivalents donor, thus maintaining a high cytosolic GSH:GSSG ratio of 30 – 100:1 [15,16]. This provides a reducing intracellular environment, which is thought to retain the oxidation-sensitive thiol groups of cysteine residues of proteins in a reduced state [17]. Overexpression of glutathione reductase from *Oryza sativa* and *Brassica rapa* in *S. cerevisiae* has recently been shown to increase tolerance against oxidative stress induced by H_2_O_2_ and abiotic stresses such as heavy metals [18,19].

**Figure 1:**
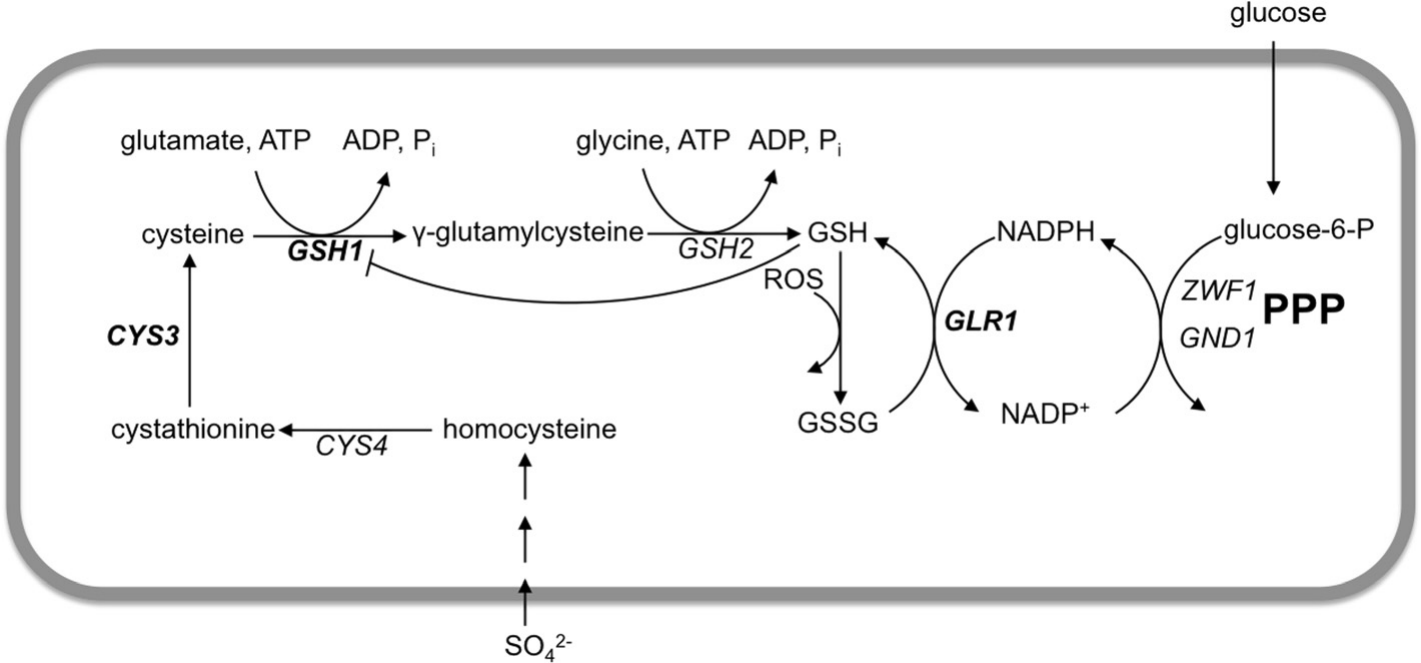
**Simplified illustration on glutathione metabolism in *S. cerevisiae *.** The genes overexpressed in the present study are marked in bold. For simplicity, only the major route for cytosolic NADPH production – PPP – is shown.

GSH is synthesized in two consecutive ATP-dependent reactions, outlined in Figure 1. The first step, catalyzed by γ-glutamylcysteine synthetase encoded by *GSH1*, has shown to be rate-limiting as overexpression of *GSH2* led to unchanged levels of total glutathione, whereas overexpression of *GSH1* resulted in an almost twofold increase in the intracellular GSH levels [20]. Yeast strains overexpressing *GSH1* have been shown to possess higher tolerance to oxidative stress induced by H_2_O_2_ compared to wild type cells [21]. In addition to overexpression of *GSH1*, supplementation of the constituent amino acids of GSH, and in particular cysteine, has shown to increase GSH accumulation in *S. cerevisiae*[22]. Increased expression of *CYS3*, encoding cystathionine-γ-lyase, was found in a UV-mutagenized strain of *S. cerevisiae*, which accumulated high levels of GSH [23].

In the present study, we investigated if engineering of the cell’s antioxidant system by overexpression of three genes (*GSH1, CYS3* and *GLR1*) involved in the metabolism of the endogenous antioxidant glutathione could alleviate the toxic effects induced by lignocellulose-derived inhibitors, and thereby enhance robustness of *S. cerevisiae* for lignocellulosic hydrolysate fermentation. After confirming that the recombinant strains had altered redox characteristics by quantifying the intracellular glutathione levels, we show that the strains engineered for higher intracellular glutathione levels have superior robustness under process-like conditions in a simultaneous saccharification and fermentation (SSF) setup.

## Results

Several inhibitors generated during pretreatment of lignocellulosic biomass are known to influence the redox balance of the fermenting organism [24]. In the present study, the redox state of *S. cerevisiae* was modified by modulating the intracellular levels of glutathione by overexpressing three genes involved in glutathione biosynthesis and metabolism: *GSH1, CYS3* and *GLR1* (Figure 1). The mutant strains were then evaluated in simultaneous saccharification and fermentation of spruce.

### Growth performance and intracellular glutathione concentration of recombinant strains

The strains constructed in the present study are shown in Table 1. The genes were put under the control of strong constitutive promoters of either the *TDH3* or *TPI1* genes and integrated into the genome of CEN.PK background strains. The maximum specific growth rate in mineral medium containing glucose as sole carbon source of the resulting strains is showed in Table 2. In these conditions, no dramatic differences in specific growth rate were observed among the strains. CEN.PK 113-7D (wild type) showed the highest specific growth rate of 0.40 ± 0.01 h^-1^, whereas the strain overexpressing *GSH1/GLR1* showed the lowest specific growth rate of 0.34 ± 0.00 h^-1^. All strains overexpressing *GSH1* had a lower specific growth rate than the wild type whereas *GLR1* overexpression alone did not influence growth kinetics in defined mineral medium.

**Table 1:** Strains used and constructed in the study

**Strain background**	**Recombinant strain**	**Genotype**	**Reference**
CEN.PK 113-7D	-	*MATa, MAL2-8*^*c*^*, SUC2*	[25]
CEN.PK 113-5D	*GSH1*	*ura3-52*::*URA3-TDH3p-GSH1-CYC1t, MATa, MAL2-8*^*c*^*, SUC2*	This study
CEN.PK 102-3A	*GSH1/CYS3*	*ura3-52*::*URA3-TDH3p-GSH1-CYC1t, leu2-3,112*::*LEU2-TPI1-CYS3-CYC1t, MATa, MAL2-8*^*c*^*, SUC2*	This study
CEN.PK 113-3C	*GLR1*	*trp1-289*::*TRP1-TDH3p-GLR1-CYC1t, MATa, MAL2-8*^*c*^*, SUC2*	This study
CEN.PK 113-9D	*GSH1/GLR1*	*ura3-52*::*URA3-TDH3p-GSH1-CYC1t, trp1-289*::*TRP1-TDH3p-GLR1-CYC1t, MATa, MAL2-8*^*c*^*, SUC2*	This study
CEN.PK 113-6B	*GSH1/CYS3/GLR1*	*ura3-52*::*URA3-TDH3p-GSH1-CYC1t, leu2-3,112*::*LEU2-TPI1-CYS3-CYC1t, trp1-289*::*TRP1-TDH3p-GLR1-CYC1t MATa, MAL2-8*^*c*^*, SUC2*	This study

**Table 2:** Maximum specific growth rate and intracellular concentrations of total, reduced and oxidized glutathione in the strains in this study

**Strain**	**μ**_**max**_**(h**^**-1**^**)**	**Total glutathione (μmol g DW**^**-1**^**)**	**GSH (μmol g DW**^**-1**^**)**	**GSSG (μmol g DW**^**-1**^**)**	**E**_**hc**_**(mV)**	**[GSH]:[GSSG]**
CEN.PK 113-7D	0.40 ± 0.01	8.8 ± 0.7	8.7 ± 0.7	0.10 ± 0.01	-225 ± 2	88 ± 10
*GSH1*	0.36 ± 0.01	12.5 ± 2.0	12.2 ± 2.0	0.33 ± 0.03	-218 ± 5	37 ± 8
*GSH1/CYS3*	0.37 ± 0.00	12.5 ± 0.3	12.1 ± 0.3	0.38 ± 0.04	-216 ± 1	32 ± 3
*GLR1*	0.39 ± 0.00	8.9 ± 1.1	8.8 ± 1.1	0.13 ± 0.01	-221 ± 4	67 ± 9
*GSH1/GLR1*	0.34 ± 0.00	11.3 ± 1.4	11.0 ± 1.4	0.29 ± 0.03	-217 ± 2	37 ± 1
*GSH1/CYS3/GLR1*	0.37 ± 0.01	12.2 ± 0.3	12.0 ± 0.2	0.19 ± 0.03	-225 ± 1	62 ± 7

The values given are mean values of triplicate cultivations ± standard deviation.

**Figure 2:**
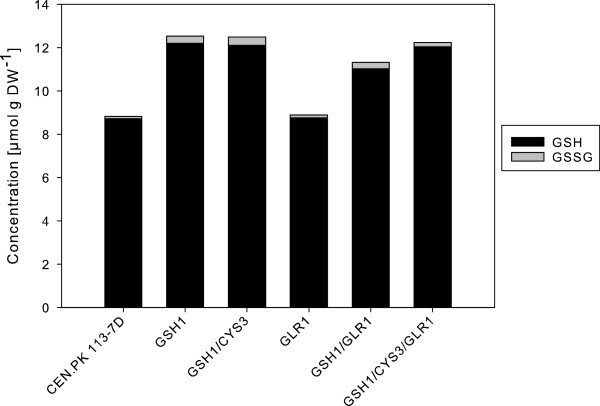
**Intracellular levels of reduced (GSH) and oxidized (GSSG) glutathione.** The bars represent mean values from triplicate cultivations.

To assess the effect of overexpression of *GSH1, CYS3* and *GLR1* on intracellular glutathione levels, total and oxidized glutathione were quantified in early exponential phase in cells grown in defined mineral medium (Figure 2). The concentration of GSH could then be deduced from the measured amounts. The strains overexpressing *GSH1* and *GSH1/CYS3* showed the highest increase in total glutathione concentration, accumulating 42% higher levels than the wild type. The oxidized glutathione levels (GSSG) were also higher for these recombinant strains. When *GSH1/CYS3/GLR1* were overexpressed, a 39% increase in total glutathione was observed, whereas overexpression of *GSH1/GLR1* resulted in a 28% increase in the total glutathione levels. Overexpression of *GLR1* alone did not alter the levels of total GSH compared to the wild type. The results are summarized in Table 2.

The intracellular levels of glutathione can be used to characterize the state of the intracellular redox environment. Two principal approaches have been used for this purpose: the [GSH]:[GSSG] ratio and the GSSG/2GSH half-cell potential (E_hc_).

The [GSH]:[GSSG] ratio varied considerably among the strains (Table 2). The highest ratio was observed in the wild type, where it was estimated to be 88. Overexpression of *GSH1* resulted in a decrease of the ratio to 37. Although glutathione reductase catalyzes the conversion of GSSG to GSH, overexpression of this gene did not result in a higher [GSH]:[GSSG] ratio than the wild type. In fact, the ratio was estimated to 67, thus 24% lower than the value for CEN.PK 113-7D. Surprisingly, *GSH1* overexpression in combination with *GLR1* did not change the ratio compared to the strain where only *GSH1* was overexpressed, and consequently it remained at a low level compared to the wild type strain. The lowest ratio (i.e. 32) was observed in the strain overexpressing *GSH1* and *CYS3.* When all three genes were overexpressed in the same strain, the [GSH]:[GSSH] ratio was restored to a similar level as the strain overexpressing only *GLR1*, but the ratio was still significantly lower than for the wild type.

E_hc_ can be calculated from the Nernst equation after determination of the intracellular concentrations of reduced and oxidized glutathione (see methods section). The estimated E_hc_ values are listed in Table 2. The half-cell redox potentials for the strains overexpressing *GSH1/GLR1* and *GSH1*/*CYS3* were significantly higher than the wild type, indicating a more oxidizing environment in these strains. The E_hc_ values estimated for the other strains were not statistically different from the wild type.

### Evaluation of strains in simultaneous saccharification and fermentation

**Figure 3:**
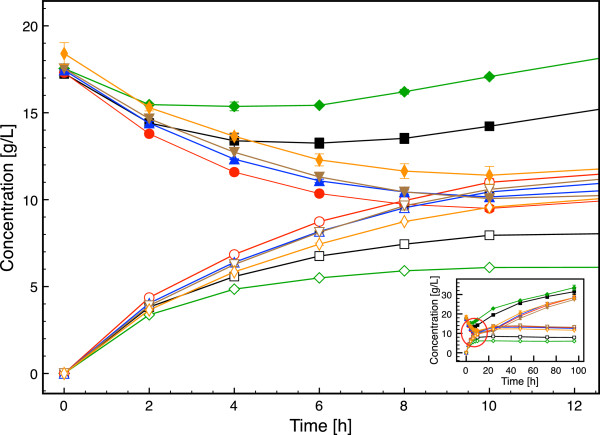
**Time course of glucose and ethanol concentrations in SSF of spruce with the strains investigated in the present study.** Black squares: CEN.PK 113-7D, red circles: *GSH1*, blue triangles: *GSH1/CYS3*, green diamonds: *GLR1*, brown reversed triangles: *GSH1/GLR1*, orange stretched diamonds: *GSH1/CYS3/GLR1*. The filled and open symbols represent glucose and ethanol, respectively. The data points are mean values from two independent cultivations. The error bars represent the maximum and minimum values.

Simultaneous saccharification and fermentation (SSF) has been proposed as an attractive process concept for lignocellulosic bioethanol production [26,27]. In this process, the enzymatic hydrolysis occurs concomitantly with the fermentation in the same reaction vessel. The advantages result from the low end-product inhibition of cellulolytic enzymes, the short process time and the lower investment costs compared to separate hydrolysis and fermentation (SHF) [27]. The recombinant strains constructed in the present study were evaluated in SSF of pretreated spruce, to investigate if the engineering strategy resulted in increased robustness at process-like conditions. Spruce was chosen as it has been shown to be one of the most challenging materials in terms of inhibitor content [28,29]. As the cellulolytic enzymes generally require higher temperatures than the microbes, the process was carried out at 35°C. The composition of the pretreated raw material is shown in Table 3 and a time course of glucose and ethanol concentrations during the SSF are shown in Figure 3. All strains started to consume glucose and produce ethanol instantly after inoculation. All recombinant strains with increased intracellular glutathione levels, that is *GSH1, GSH1/CYS3, GSH1/GLR1* and *GSH1/CYS3/*GLR1, sustained ethanol production for 10 hours, with consistent decrease in free glucose concentration (Figure 3). In contrast, glucose started to accumulate in the cultures with the strain overexpressing *GLR1* and the wild type after 4 and 6 hours respectively, as a result of the sugar release rate from the lignocellulosic material superseding the glucose consumption rate of the microorganism. As a consequence, the highest ethanol concentration was achieved in the cultivation with the strain overexpressing both *GSH1* and *GLR1*, which reached 14.0 g L^-1^ ethanol after 48 hours. The strains overexpressing *GSH1*, *GSH1/CYS3* and *GSH1/CYS3/GLR1* produced 13.7, 13.1 and 12.3 g L^-1^ ethanol, respectively, after 48 hours. The lowest maximal ethanol concentration was achieved in the cultivation with the strain overexpressing *GLR1* (6.0 g L^-1^ at 48 hours), followed by the wild type that reached 8.2 g L^-1^ at 48 hours. The maximum ethanol concentrations achieved and the ethanol yields on hexoses are reported in Table 4.

**Table 3:** Composition of pretreated spruce at 10% (w/w) WIS

**Component**	**Concentration [g L**^**-1**^**]**
**Solid fraction**	
Glucose	51.9
Mannose	0.3
Xylose	0.2
**Liquid fraction**	
Glucose	17.6
Mannose	11.6
Xylose	5.2
Galactose	2.4
Arabinose	1.6
Acetic acid^a^	3.3
Formic acid^a^	0.1
Levulinic acid^a^	0.8
HMF^a^	2.3
Furfural^a^	1.4

^a^Compounds that are classified as inhibitors of growth and ethanol production in yeast.

**Table 4:** Results from SSF of pretreated spruce with the strains constructed in the present study

**Strain**	**Ethanol yield on hexoses**^**a**^**(g g**^**-1**^**)**	**Ethanol concentration at 48 h (g L**^**-1**^**)**
CEN.PK 113-7D	0.10 ± 0.00	8.2 ± 0.0
*GSH1*	0.16 ± 0.00	13.7 ± 0.2
*GSH1/CYS3*	0.16 ± 0.00	13.1 ± 0.3
*GLR1*	0.07 ± 0.00	6.0 ± 0.0
*GSH1/GLR1*	0.17 ± 0.01	14.0 ± 1.0
*GSH1/CYS3/GLR1*	0.14 ± 0.00	12.3 ± 0.2

^a^The ethanol yield was calculated based on the total amount of hexoses present in the solid and liquid fraction of the pretreated raw material at the start of the SSF.

The values given are mean values from duplicate cultivations ± standard deviation.

**Figure 4:**
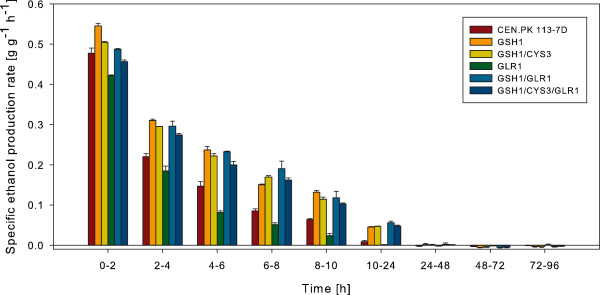
**Specific ethanol production rates over time in SSF of spruce with the strains investigated in the present study.** The specific rates were calculated assuming a cell concentration of 4 g L^-1^ throughout the whole fermentation course. The bars represent mean values from two independent cultivations. The error bars show the maximum and minimum values.

In order to study how the specific production rate of ethanol varied during the course of the fermentation, this parameter was calculated between each sampling point (Figure 4). The specific production rate of ethanol decreased during the course of the process, and was equal to 0 after 48 hours of fermentation for all strains. The highest initial production rate was observed in the cultivation with the strain overexpressing *GSH1* between 0 and 2 hours. In general, all strains harboring the *GSH1* overexpression construct maintained a higher specific production rate of ethanol than the wild type and the *GLR1* overexpressing strain during the first 48 hours of cultivation.

**Figure 5:**
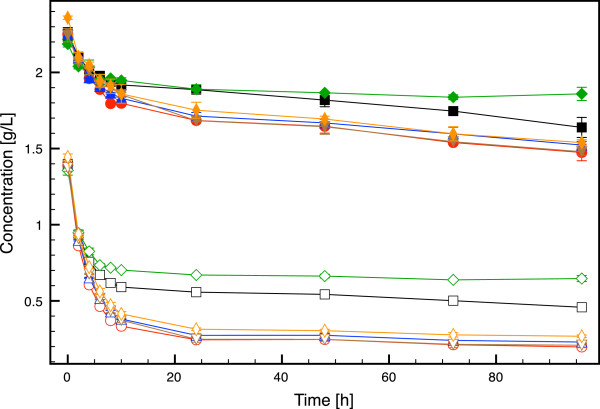
**Time course of HMF and furfural concentrations in SSF of spruce with the strains investigated in the present study.** Black squares: CEN.PK 113-7D, red circles: *GSH1*, blue triangles: *GSH1/CYS3*, green diamonds: *GLR1*, brown reversed triangles: *GSH1/GLR1*, orange stretched diamonds: *GSH1/CYS3/GLR1*. The filled and open symbols represent HMF and furfural, respectively. The data points are mean values from two independent cultivations. The error bars represent the maximum and minimum values.

Both HMF and furfural were converted to different extent by the investigated strains in the SSF cultivation performed in the present study (Figure 5). The conversion of both furan aldehydes were active as long as the cells were viable and consumed sugars. All four strains overexpressing *GSH1* converted the largest amount of HMF and furfural during the course of the cultivation, thus outperforming the wild type strain. Similar results were also obtained in cultivations using a synthetic hydrolysate (data not shown). The strain overexpressing *GLR1* converted the lowest amount of HMF and furfural in the present study.

## Discussion

In the present work we show that by increasing the intracellular concentration of glutathione, strain robustness could be improved in industrially relevant conditions posed by pretreated spruce in an SSF process. As a mechanism for the improved robustness, we hypothesize a redox buffering capacity potentiating effect, originating from the increased availability of reduced glutathione. The enhanced redox buffering capacity would confer higher inherent capability of the intracellular environment to titrate cofactor consuming exogenous molecules. Indeed, at the time of writing this article, it was reported that furan aldehydes, and in particular furfural, depleted the intracellular pool of glutathione. This fact further corroborates the hypothesis of the redox potentiating effects of increasing the intracellular concentration of GSH [11]. In the same paper, it was shown that overexpression of *GSH1* resulted in a decreased lag-phase of growth in the presence of furfural, whereas no effect was observed in the case of HMF. In fact, similar strategies, such as overproduction of the intracellular protective metabolite and ROS scavenger ascorbic acid, have been shown to increase robustness to various environmental stresses [30]. It is known that inhibitors such as HMF and furfural not only drain microorganisms of reducing power, but have also been shown to induce the generation of reactive oxygen species in *S. cerevisiae*[5,10]*.*

Quantification of the [GSH]:[GSSG] ratio and the half-cell redox potential E_hc_ were used to assess the magnitude of the changes in intracellular redox environment upon overexpressing genes involved in the glutathione biosynthesis pathway. E_hc_, which is calculated from the Nernst equation (see Methods section), has been reported to be a more reliable method, as the stoichiometry of the glutathione redox couple (GSSG + 2H^+^+2e^-^ → 2GSH) is taken into account [13,15,31]. E_hc_ is thus dependent on both the [GSH]:[GSSG] ratio and the absolute concentration of GSH, which means that cells having the same [GSH]:[GSSG] ratios can have different redox potentials depending on the concentration of GSH. Consequently, cells with a high concentration of intracellular reduced glutathione have a higher buffering capacity against oxidative insults than cells with a lower concentration of reduced glutathione.

Although overexpression of *GSH1* increased the intracellular levels of reduced glutathione, unexpectedly none of the recombinant strains had a more reducing intracellular environment compared to the wild type when grown in defined mineral medium (as it would be indicated by a higher [GSH]:[GSSG] ratio and/or a lower E_hc_). In fact, the ultimate reason for the higher estimates of E_hc_ in these strains was indeed the higher intracellular concentration of GSSG in all recombinant strains harboring the *GSH1* overexpression construct. However, it has been shown in a recent study [32] that the cytosolic glutathione redox potential was independent of changes in whole-cell GSSG levels due to compartmentalization of GSSG. The GSSG concentration was maintained at very low levels in the cytosol through the action of Ycf1p pumping GSSG to the vacuole. Hence, calculating the E_hc_ using GSSG levels determined from whole cell-extracts as in the present study in fact overestimates the cytosolic redox potential. Moreover, it has recently been demonstrated that significant differences exist in the redox potential between different cellular compartments [33]. At a purely speculative level, assuming an oxidation level of 0.03% of the cytosolic glutathione pool (as estimated by Østergaard et al. [34]) for all strains, the estimated E_hc_ values for all recombinant strains overexpressing *GSH1* would in fact be lower than those in the wild type (-277 mV versus -273 mV), which then would indicate that *GSH1* overexpression increases the redox buffering capacity and consequently strain robustness.

The increased redox buffering capacity of the strains overexpressing *GSH1* was evident in SSF of pretreated spruce at 10% (w/w) water insoluble solids (WIS) concentration, which contains a spectrum of inhibitory compounds and in particular significant amounts of HMF and furfural (Table 3) [24]. All strains carrying the *GSH1* overexpression construct were able to consume glucose and convert HMF and furfural for a longer period of time resulting in higher ethanol yields for these strains than the wild type. As overexpression of *CYS3* in combination with *GSH1* did not result in higher intracellular concentrations of glutathione than overexpression of *GSH1* alone, the performance of this strain did not differ form the latter. An explanation could be that the high GSH levels obtained by *GSH1* overexpression prevent a further increase of the intracellular glutathione concentration, since it is known that Gsh1p is feedback inhibited by GSH [35]. Unexpectedly, overexpression of *GLR1* in combination with *GSH1* did not result in decreased levels of GSSG, possibly due to limitation of NADPH. Consequently, the *GSH1/GLR1* strain performance was equivalent to that of the strain overexpressing *GSH1*. In contrast, *GLR1* overexpression alone was found to be a burden for the cells when cultivated in the spruce slurry, which was manifested in an earlier cessation of ethanol production than the wild type. Competition for NADPH between Glr1p and furan aldehyde detoxifying oxidoreductases could be a possible explanation for this observation.

Although integration of the overexpression constructs influenced the maximum specific growth rate, the difference between the strains was not severe. All recombinant strains carrying the *GSH1* overexpression construct exhibited a lower specific growth rate than the wild type, and the strain overexpressing both *GSH1* and *GLR1* grew at the lowest specific growth rate. The lower specific growth rates can be explained by a combination of metabolic burden on the cell imposed by the overexpression of the introduced genetic constructs, and uncharacterized effects on strain growth behavior commonly encountered as a consequence of using auxotrophic markers. There is also a possibility that the decrease in specific growth rate in the strains overexpressing *GSH1* is connected to partial depletion of cysteine due to overproduction of glutathione. In fact, overexpression of *CYS3* somewhat restored the specific growth rate in strains overexpressing *GSH1* (Table 2). On the other hand, these small changes in specific growth rate may be of minor importance for industrial bioethanol production, since the microorganisms are thought to reside in a non-growing state in a process such as SSF [36]. Therefore, an engineering strategy that results in a small decrease in specific growth rate may not be unfavorable as long as the microorganism is more tolerant to the process conditions.

Overall, the results from this study show that engineering of *S. cerevisiae* with increased intracellular glutathione production has a beneficial effect on strain robustness by extending the time of survival in an SSF process using a challenging substrate. The mechanism for the increased tolerance supposedly originates from an increased redox buffering capacity resulting from the increased pool of reduced glutathione in the recombinant strains.

## Conclusions

In this work, we showed that engineering of the redox metabolism of *S. cerevisiae* in terms of increasing the intracellular levels of glutathione by overexpressing *GSH1* resulted in increased strain robustness in an SSF process. This was reflected in higher cell survival and final ethanol concentrations of the recombinant strains compared to the wild type in industrial media.

## Methods

### Strains

The strains used in the present study are listed in Table 1. CEN.PK 113-7D (*MATa, MAL2-8*^*c*^*, SUC2)* was used as reference strain.

### Plasmid construction

*GSH1* was amplified from genomic DNA of CEN.PK 113-7D using High-Fidelity DNA Polymerase (Thermo Fisher Scientific) with primers GSH1_SalI_fw (5′ ATAAGTCGACATGGGACTCTTAGCTTTGGG) and GSH1_KpnI_rev (5′ GCATGGTACCTTAACATTTGCTTTCTATTGAAG) and cloned into the integrative plasmid YIplac211 [37] harboring the promoter *TDH3p,* the terminator *CYC1t* and a functional copy of *URA3. CYS3* was codon-optimized with JCat [38] and ordered sub-cloned into YIplac128 (containing a functional copy of *LEU2)* with *TPI1p* and *CYC1t* from Genscript (Piscataway, NJ, USA). *GLR1* was amplified from genomic DNA of CEN.PK 113-7D with High-Fidelity DNA Polymerase (Thermo Fisher Scientific) with primers GLR1_XbaI_fw (5′TCTAGAATGCTTTCTGCAACCAAACAAAC) and GLR1_SmaI_rev (5′ ACCCGGGTCATCTCATAGTAACCAATTCTTC) and cloned into the integrative plasmid YIplac204 harboring *TDH3p*, *CYC1t* and a functional copy of *TRP1*. The constructs were verified by DNA sequencing.

### Strain construction

All yeast transformations were performed according to [39]. CEN.PK 113-5D, CEN.PK 102-3A, CEN.PK 113-9D and CEN.PK 113-6B were transformed with YIplac211-*TDH3p-GSH1-CYC1t* previously linearized in *URA3* with StuI. Selection was done on Yeast Nitrogen Base (YNB) plates with addition of relevant amino acids when applicable. CEN.PK 102-3A and CEN.PK 113-6B containing the overexpression construct for *GSH1* were subsequently transformed with YIplac128-*TPIp-CYS3-CYC1t* previously linearized in *LEU2* with ClaI and selected on YNB agar plates with supplementation of amino acids when applicable. CEN.PK 113-3C, CEN.PK 113-9D (containing *GSH1* overexpression construct) and CEN.PK 113-6B (containing *GSH1* and *CYS3* overexpression constructs) were transformed with YIplac204-*TDH3p-GLR1-CYC1t* previously linearized in *TRP1* with BstXI and selected on YNB agar plates with supplementation of amino acids when applicable. All integrations were verified by PCR.

### Specific growth rate determination and quantification of intracellular glutathione

Single colonies of the respective strains were picked from YPD plates and inoculated in 5 mL defined mineral medium according to [40] containing 20 g L^-1^ glucose and 50 mM potassium hydrogen phthalate. After 24 hours, 50 mL medium of the same composition was inoculated at a final OD_600_ of 0.2. Samples were taken at regular intervals during the exponential phase and the maximum specific growth rate was determined from the slope of ln(OD_600_) plotted against time (h). The final OD_600_ at the end of the exponential phase was approximately 5. In early exponential phase (when OD_600_ was 1.1-1.5), 5 mL samples were withdrawn and centrifuged at 2400 g for 4 minutes at 4°C. Total glutathione was determined according to [32,41]. The cells were washed with 0.1 M potassium phosphate buffer, pH 7.5, resuspended in ice-cold 8 mM HCl, 1.3% (w/v) 5-sulfosalicylic acid solution and disintegrated with glass beads (particle size 425–600 μm) in a bead mill for 3*30 s at maximum speed. The lysate was centrifuged at 10 000 g for 5 minutes at 4°C and the supernatant was used for total glutathione estimation. Samples for oxidized glutathione quantification were prepared by letting 100 μL extract of the preparation above react with 2 μL 20% (v/v) 2-vinylpyridine in ethanol. The pH was brought to 7 by addition of 40 μL 1 M Mes/Tris buffer at pH 7 and the reaction took place at room temperature for 1 hour. Final concentrations in the reaction mix were the following: 1.2 IU/ mL GSH reductase; 0.73 mM DTNB; 0.24 mM NADPH; 0.09% 5-sulfosalicylic acid. Standard curves were constructed using reduced glutathione or oxidized glutathione, respectively, ranging from 0.206 to 26.4 μM .The assay was performed in 96-well plates in a Fluostar microplate reader (BMG Labtech GmbH, Offenburgh, Germany) reading absorbance at 412 nm after automatic addition of NADPH to start the reaction. The absorbance was read every 5 s for 2 min in each reaction.

### Calculation of intracellular redox environment

The intracellular redox environment was estimated from the Nernst equation according to [13] using the intracellular concentrations of GSH and GSSG. An intracellular volume of 2.38 mL gDW^-1^ was assumed when calculating the intracellular concentrations [42] (visual inspection under microscope did not reveal any significant differences in cell size between the strains).

$$E_{\mathrm{hc}}=E_{0}-2.303\frac{\mathrm{RT}}{\mathrm{nF}}\log_{10}\frac{{\left[\mathrm{GSH}\right]}^{2}}{\left[\mathrm{GSSG}\right]}$$

where E_0_ is the standard potential for GSH (-240 mV) at pH 7 [43], R is the gas constant (8.31 J mol^-1^ K^-1^), T is the absolute temperature (303 K), n is the number of electrons exchanged in the process (2) and F is the Faraday constant (96485 C mol^-1^).

### Simultaneous saccharification and fermentation (SSF)

Precultures were prepared by inoculating a single colony of the respective strain in 5 mL defined mineral medium according to [40] containing 50 g L^-1^ glucose and twice the amounts of the other components. After 24 hours, the first preculture was harvested and inoculated at a final OD of 0.2 in the above mentioned medium. The second preculture was harvested after all glucose had been consumed, which was monitored with glucose test strips (Keto-Diabur-Test 5000, Roche, Basel, Switzerland). SSF was carried out at 50 g working weight in shake-flasks with steam-pretreated spruce provided by SEKAB E-Technology (Örnsköldsvik, Sweden) at 10% (w/w) water insoluble solids (WIS) content. The slurry was supplemented with 1 g L^-1^ yeast extract, 0.5 g L^-1^ (NH_4_)_2_HPO_4_, 0.025 g L^-1^ MgSO_4_^.^7H_2_O and 50 mM potassium phthalate. The SSF experiment was initiated by addition of cell suspension yielding a final cell concentration of 4 g L^-1^ DW and by addition of Celluclast 1.5 L (Novozymes A/S, Bagsvaerd, Denmark) and Novozyme 188 (Novozymes A/S, Bagsvaerd, Denmark) at 10 mg protein/g WIS and 500 nkat/g WIS, respectively. Samples were withdrawn regularly throughout the cultivations, which were performed in duplicates at 35°C and pH 5.

### Quantification of sugars and extracellular metabolites

Samples from SSF experiments were centrifuged at 14000 g for 2 minutes and filtered through 0.2 μm nylon membranes. The samples were stored at -20°C until analysis. Concentrations of glucose, xylose, mannose, galactose, ethanol, glycerol, xylitol, HMF and furfural were analyzed using an HPLC system (Dionex, Sunnyvale, CA) equipped with an Aminex HPX87-P column (Bio-Rad Laboratories, Munich, Germany) operated at 85°C with milliQ-H_2_O as eluent at a flow rate of 0.6 mL min^-1^. Detection of sugars and alcohols were made with a Shodex RI-101 refractive index detector (Showa Denko, New York, NY). HMF and furfural were detected with an UV detector at 210 nm (Dionex, Sunnyvale, CA).

### Determination of cell mass

Cell mass was determined according to [44]. 5 mL of culture broth was filtered through a 0.45 μm PES membrane (Sartorius Stedim, Aubagne, France). The filters were washed with MilliQ-H_2_O and dried in a microwave oven at 120 W for 15 minutes and subsequently weighed.

## Competing interests

The authors declare that they have no competing interests.

## Authors’ contributions

MA participated in the design of the study, constructed the strains, performed the experiments and wrote the manuscript. VM participated in the design of the study, participated in the strain construction and commented on the manuscript. HH participated in the strain construction. LO participated in the design of the study and commented on the manuscript. MB participated in the design of the study and commented on the manuscript. All authors read and approved the final manuscript.
